# Supplementary material for: Individualized Perioperative Hemodynamic Management Using Hypotension Prediction Index Software and the Dynamics of Troponin and NTproBNP Concentration Changes in Patients Undergoing Oncological Abdominal Surgery
Source: J Pers Med. 2024 Feb 16;14(2):211. doi: 10.3390/jpm14020211 (PMC10890224; doi:10.3390/jpm14020211)
Supplement: Supplementary file 1 [file jpm-14-00211-s001.zip › jpm-2850638-supplementary.pdf]

Supplementary material

Supplementary Table S1. The changes in NTproBNP levels over a 48-hour period post-surgery and the co-occurrence of chronic diseases.

Variable	Specific comorbidity - YES			Specific Comorbidity-NO					
	Mean rank	*Mdn*	*IQR*	Mean rank	*Mdn*	*IQR*	*Z*	*p*	*r*
				NT					
Δ NT 2-0	24,36	38,25	361,52	23,13	87,25	142,32	-0,29	0,775	0,04
%Δ NT 2-0	27,64	239,75	281,56	21,69	150,18	131,83	-1,38	0,166	0,20
				CHNS					
Δ NT 2-0	21,93	38,25	135,60	26,44	109,70	562,82	-1,08	0,278	0,16
%Δ NT 2-0	23,73	171,42	189,38	23,06	159,60	264,95	-0,16	0,872	0,02
				Astma					
Δ NT 2-0	23,05	44,80	241,50	30,00	84,40	44,30	-0,87	0,386	0,13
%Δ NT 2-0	23,23	164,85	181,30	27,33	175,66	39,14	-0,51	0,609	0,08
				Cukrzyca					
Δ NT 2-0	22,35	39,50	136,50	25,87	84,40	274,60	-0,83	0,405	0,12
%Δ NT 2-0	23,84	167,18	272,41	22,80	164,85	143,00	-0,25	0,806	0,04
				Miażdżyca					
Δ NT 2-0	20,67	22,30	137,80	27,53	107,40	242,60	-1,71	0,088	0,25
%Δ NT 2-0	23,63	187,64	240,12	23,32	164,85	159,06	-0,08	0,938	0,01
				Otyłość					
Δ NT 2-0	23,81	39,50	475,50	22,87	90,10	128,90	-0,22	0,824	0,03
%Δ NT 2-0	24,23	186,05	272,41	22,00	154,35	119,50	-0,53	0,598	0,08
				PNS					
Δ NT 2-0	22,68	37,00	192,60	24,48	90,10	290,35	-0,45	0,651	0,07
%Δ NT 2-0	25,60	226,99	269,58	21,00	153,10	145,86	-1,16	0,247	0,17
				PNN					
Δ NT 2-0	24,81	69,10	304,62	18,80	62,25	730,70	-1,25	0,211	0,18
%Δ NT 2-0	25,47	186,85	268,31	16,40	133,40	134,37	-1,89	0,059	0,28

Abbreviations : ΔNT-pro-BNP 2-0: Change in NT-pro-BNP concentration between the second day and the preoperative measurement,
%ΔNT-pro-BNP 2-0: Percentage change in NT-pro-BNP concentration between the second day and the preoperative measurement.

Supplementary Table S2. Correlations between the change in NT-proBNP between the preoperative level and the second day post-operation, and the proportions between these levels and age.

	ΔNT-pro-BNP 2-0		%ΔNT-pro-BNP 2-0:	
	r_s	*p*	r_s	*p*
ASA	0,16	0,283	-0,16	0,294
wiek	**0,38**	**0,008**	0,22	0,140
POSSUM - fizjologiczne	0,02	0,919	-0,19	0,204
POSSUM - chirurgiczne	-0,21	0,156	-0,08	0,582
POSSUM - chorobowość	-0,12	0,417	-0,20	0,193
POSSUM - śmiertelność	-0,13	0,378	-0,20	0,175
troponina	0,21	0,167	-0,03	0,855

Supplementary Table S3. Comparison of hypotension parameters depending on the baseline troponin level

	Troponin								
	No elevated (n = 27)			Above 99th percentile of lab norm (n = 18)					
Variable	średnia ranga	*Mdn*	*IQR*	średnia ranga	*Mdn*	*IQR*	*Z*	*p*	*r*
Total duration of monitoring (min)	23,61	265,00	97,00	22,08	258,50	232,75	-0,38	0,702	0,06
Number of episodes with HPI >85	21,39	9,00	10,00	25,42	14,00	13,00	-1,01	0,313	0,15
The number of hypotension episodes	20,39	1,00	2,00	26,92	2,00	3,50	-1,67	0,095	0,25
Total duration of hypotension episodes, minutes	20,94	3,00	6,00	26,08	5,00	7,75	-1,29	0,196	0,19
The proportion of time spent in hypotension, %	20,48	0,84	1,81	26,78	1,85	3,04	-1,58	0,114	0,24
Average duration of each hypotension episode, minutes	21,70	2,00	2,00	24,94	1,95	1,60	-0,81	0,416	0,12
Mean MAP if < 65mmHg	19,69	60,14	3,27	19,26	60,00	3,44	-0,12	0,907	0,02
AUT < 65mmHg	21,28	22,67	58,67	25,58	28,17	58,84	-1,08	0,280	0,16
TWA MAP < 65mmHg	20,80	0,07	0,15	26,31	0,12	0,27	-1,38	0,167	0,21
Number of episodes with MAP<50mmHg	22,70	0,00	0,00	23,44	0,00	0,00	-0,29	0,769	0,04

Supplementary table S4. Comparison of NT-proBNP parameters depending on meeting the PMI criterion.

	PMI								
	No (n = 41)			YES (n = 5)					
Variable	Mean rank	*Mdn*	*IQR*	Mean rank	*Mdn*	*IQR*	*Z*	*p*	*r*
NTproBNP – preoperative	22,93	182,90	394,10	28,20	456,00	1651,85	-0,83	0,407	0,12
NTproBNP – 24 hours	22,80	273,00	412,85	29,20	1381,00	3136,75	-1,01	0,315	0,15
NTproBNP – 48 hours	23,10	271,00	537,10	26,80	1204,00	9359,25	-0,58	0,560	0,09

Supplementary table S5. Comparison of NT-proBNP parameters depending on the occurrence of AKI.

	AKI								
	NO (n = 35)			YES (n = 11)					
Zmienna zależna	Mean Rank	*Mdn*	*IQR*	Mean Rank	*Mdn*	*IQR*	*Z*	*p*	*r*
NTproBNP – preoperative	21,17	111,40	294,00	30,91	435,90	926,60	-2,10	**0,036**	0,31
NTproBNP – 24 hours	21,69	209,70	376,70	29,27	400,50	1211,20	-1,64	0,102	0,24
NTproBNP – 48 hours	22,54	271,00	559,20	26,55	315,50	1021,00	-0,86	0,388	0,13
Δ NT 1-0	24,93	92,60	168,80	18,95	30,20	348,60	-1,29	0,198	0,19
Δ NT 2-0	25,94	99,40	323,60	15,73	20,70	325,10	-2,20	**0,028**	0,32
Δ NT 2-1	26,71	-10,40	205,70	13,27	-177,00	337,50	-2,90	**0,004**	0,43
%Δ NT 1-0	25,97	181,49	201,07	15,64	125,93	172,64	-2,23	**0,026**	0,33
%Δ NT 2-0	26,60	228,47	252,77	13,64	107,65	115,35	-2,79	**0,005**	0,41
%Δ NT 2-1	24,94	90,40	76,12	18,91	74,57	21,70	-1,30	0,193	0,19

ΔNT-pro-BNP 1-0: Change in NT-pro-BNP concentration between the first day and the preoperative measurement. ΔNT-pro-BNP 2-0: Change in NT-pro-BNP concentration between the second day and the preoperative measurement. ΔNT-pro-BNP 2-1: Change in NT-pro-BNP concentration between the second day and the first day. %ΔNT-pro-BNP 1-0: Percentage change in NT-pro-BNP concentration between the first day and the preoperative measurement. %ΔNT-pro-BNP 2-0: Percentage change in NT-pro-BNP concentration between the second day and the preoperative measurement. %ΔNT-pro-BNP 2-1: Percentage change in NT-pro-BNP concentration between the second day and the first day.

Supplementary table S6. Frequency analysis along with Fisher's exact test for the relationship between the type of surgery and the occurrence of hypotension, PMI, AKI.

		Type of Surgery					
		Open		Laparoscopic			
		n	%	n	%	p	φ/V
Intraoperative Hypotension	No	4	14,3	3	17,6	1,000	0,04
	Yes	24	85,7	14	82,4		
PMI	No	26	92,9	14	82,4	0,350	0,16
	Yes	2	7,1	3	17,6		
AKI	No	21	75,0	13	76,5	1,000	0,02
	Yes	7	25,0	4	23,5		
